# Selective six spectrophotometric methods for determination of remdesivir and moxifloxacin hydrochloride for COVID-19 treatment with overlapping spectra: a comprehensive evaluation of greenness, blueness, and whiteness

**DOI:** 10.1186/s13065-025-01607-x

**Published:** 2025-08-21

**Authors:** Eman A. Madbouly, Abdalla A. El-Shanawani, Sobhy M. El-Adl, Ahmed S. Abdelkhalek

**Affiliations:** Department of Medicinal Chemistry, Faculty of Pharmacy, Zagazig University, Zagazig, Egypt

**Keywords:** COVID-19, Remdesivir, Moxifloxacin hydrochloride, Spectrophotometry

## Abstract

Selective, and green spectrophotometric methods have been developed for the simultaneous analysis of remdesivir (RDV) and moxifloxacin hydrochloride (MFX), two active agents that are being used together in COVID-19 treatment. Because of the considerable spectral overlap, six mathematical spectrophotometric approaches were applied, including ratio derivative, ratio difference, mean centering of ratio spectra, area under the curve, Q-analysis, and bivariate calibration, to allow accurate interference-free determination without preliminary separation. The proposed methods were validated as per ICH guidelines, demonstrating excellent linearity over the concentration ranges of 1–15 µg/mL for RDV and 1–10 µg/mL for MFX, with correlation coefficients exceeding 0.999. They showed high sensitivity with LODs from 0.26 to 0.92 µg/mL and LOQs from 0.27 to 0.96 µg/mL for both drugs, enabling reliable trace analysis of the studied drugs in complex matrices. The methods also applied to dosage forms and spiked human plasma provided good recoveries with minimal matrix interference, demonstrating that the methods are robust and applicable. Eco-Scale, ComplexGAPI, and AGREE gave a high score on green profiles for environmental and practical sustainability. Further, the high whiteness and blueness scores indicated that the methods met the requirements of white analytical chemistry by RGB12 and BAGI tools. Thus, these methods offer a practical, eco-conscious solution for simultaneous determination of RDV and MFX in pharmaceutical and clinical settings, contributing to better therapeutic monitoring in COVID-19 management.

## Introduction

As far as public health emergencies go, COVID-19 is regarded as the worst of the last century. COVID-19 is a respiratory infection that was spreading quickly [1] which prompted experts to seek therapy techniques to combat the rapidly progressing respiratory disease. The need for an efficient oral antiviral treatment is still a major worry due to insufficient vaccine uptake, even though many immunizations were offered to slow the transmission of the severe acute respiratory syndrome (SARS-COV-2) virus since its widespread dissemination in 2020 [2]. Employing FDA-approved drugs like remdesivir and favipiravir was the quickest and easiest option because approving a novel therapeutic for use in humans is a drawn-out process with multiple steps [3].

**Fig. 1:**
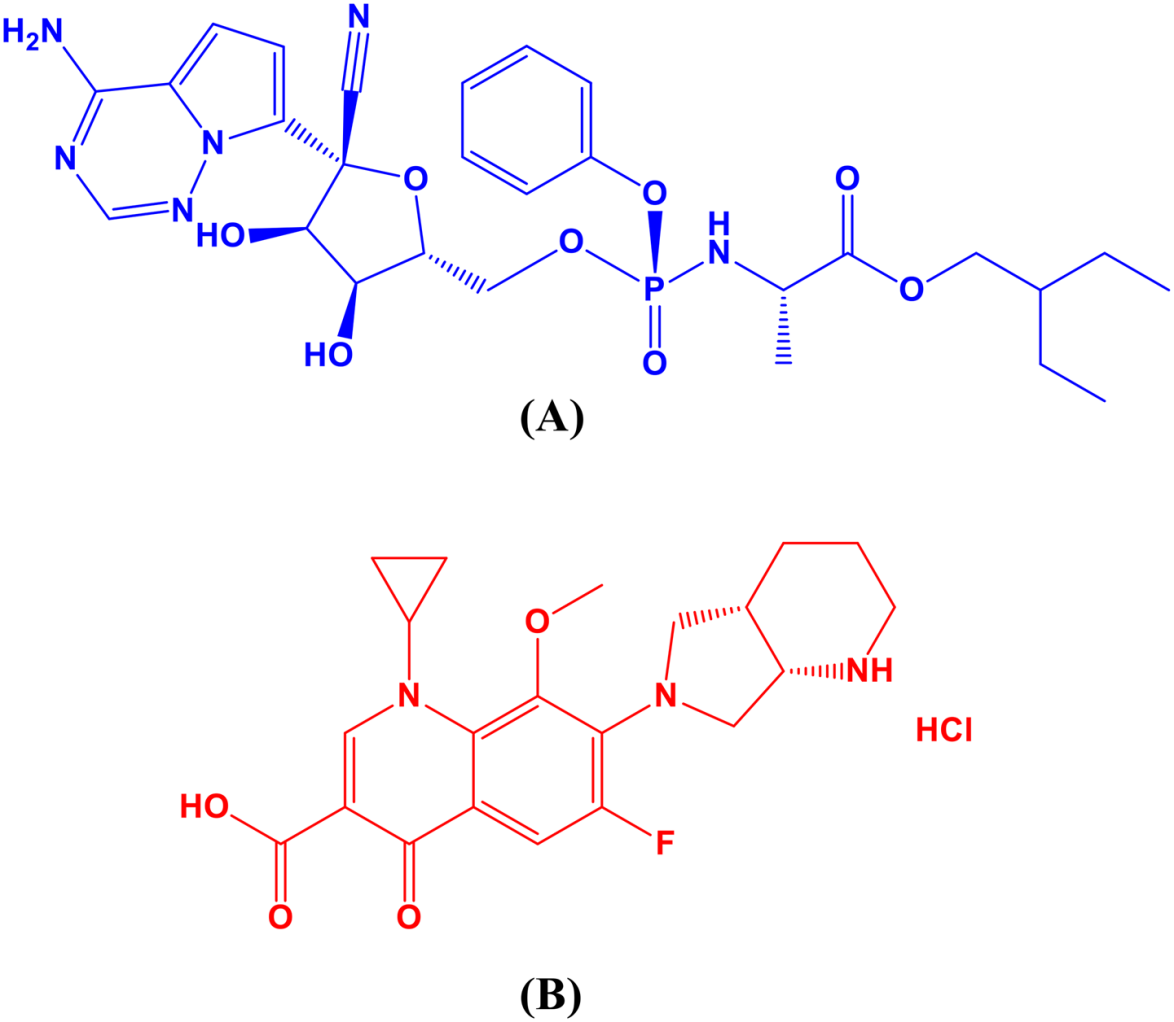
RDV (**a**) and MFX (**b**) chemical structures

Remdesivir (RDV) Fig. 1 (a), is an adenosine triphosphate analogue that has wide antiviral action. Preliminary research suggests that RDV may hasten recovery for hospitalized patients with severe COVID-19. It was the first medication that the US Food and Drug Administration (FDA) had authorized for use in an emergency to treat hospitalized COVID-19 patients [4, 5]. Antivirals function by blocking RNA polymerase, hence stopping the propagation of coronaviruses. RDV measurement has been reported using a number of techniques, including chromatographic [6–9], spectrophotometric [10–15], electrochemical [16, 17] and spectrofluorimetric [18–23].

Despite the fact that COVID-19 is a virus, there are concerns about the drug’s regular use in treating the disease. Viral respiratory infections can raise the likelihood of developing bacterial pneumonia, however medicines have no direct effect on SARS-CoV-2 [24]. A new in silico study suggests that fluoroquinolones, such moxifloxacin hydrochloride (MFX), Fig. 1(b), may prevent SARS-CoV-2 from replicating because of their high affinity for binding to the virus’s primary protease enzyme [25]. Many analytical techniques are listed in the literature review for MFX analysis, such as densitometric [26, 27], liquid chromatographic [28–33], spectrophotometric [34–36], and spectrofluorometric [37–40] approaches.

The simultaneous determination of remdesivir (RDV) and moxifloxacin (MFX) is crucial due to their frequent co-administration in COVID-19 treatment [41, 42]. Several spectrophotometric methods have been previously reported for resolving overlapping spectra of such drug combinations, including absorbance subtraction, extended ratio subtraction, and amplitude modulation [42]. These techniques have demonstrated successful applications, particularly in pharmaceutical dosage forms and even in spiked biological matrices. Nevertheless, their performance often depends on specific spectral characteristics, such as plateau regions or isoabsorptive points, and may require precise wavelength selection and mathematical manipulation.

To address these issues, the current study proposes a fully applicable spectrophotometric methodology that incorporates six mathematical techniques: ratio derivative (^1^DD), ratio difference (RD), mean centering of ratio spectra techniques (MC), area under the curve (AUC), Q-analysis, and bivariate calibration -all of which improve selectivity, sensitivity, and versatility by allowing quantification of both drugs without spectral interference. This proposed methodology is also more resilient than previously published methods because it uses less data processing and has a broader application, rather than being limited to specific wavelengths of each spectral point [43–47].

Scientific societies have recently pushed for the integration of green analytical chemistry (GAC) and white analytical chemistry (WAC) concepts into research workflows in response to the increased emphasis on comprehensive, sustainable, inventive, and validated analytical methodologies [48, 49]. Many techniques, including the ESA (Eco-Scale Assessment), GAPI (Green Analytical Procedure Index), and the AGREE (Analytical Greenness Metric) have recently been developed for evaluating “greenness” in accordance with the 12 GAC principles [50–52].

Furthermore, a variety of recently developed algorithms, including RGB12 and BAGI algorithms, have been developed for evaluating “whiteness” and “blueness” [53, 54].

## Experimental

### Instrumentation

A Shimadzu UV-Visible 1800 Spectrophotometer (Tokyo, Japan) with corresponding 10 mm quartz cells was used in the experiment. The apparatus was operated using Shimadzu’s UV-Probe personal spectroscopy software, version 2.21. Furthermore, the investigations made use of an analytical balance (Precisa125A, Switzerland).

### Chemicals

Remdesivir (99.15%) and MFX (99.45%) powders were kindly given by EVA Pharma for Pharmaceuticals and Medical Appliances (Cairo, Egypt) according to the reference methods [15, 36]. EVA Pharma (Cairo, Egypt) produced Remdesivir Eva pharma^®^ vials with 100 mg of RDV, which were purchased from the local market (batch numbers: 2009561, 2009563, 2009566, 2009567). Additionally, Moxiflox^®^ tablets, produced by EVA Pharma (Cairo, Egypt) and containing 436.37 mg of MFX (400 mg moxifloxacin) per tablet, were bought locally (batch number 2212537). Zagazig University Hospital (Zagazig, Egypt) kindly provided plasma samples, which were kept frozen at -20 °C until assessment. Sigma-Aldrich in Germany supplied methanol.

### Standard solutions

For RDV and MFX stock standards, it was necessary to dissolve 100 mg of each drug powder in 70 mL of methanol, sonicate for 15 min, and then complete to 100 mL with methanol. Then dilute the stock solutions with methanol to obtain working standard solutions (100 µg/mL).

### Laboratory prepared mixtures

The standard solutions of RDV and MFX were accurately aliquoted into 10 mL volumetric flasks, and methanol was used to dilute the solution with various ratios.

## Procedures

### Calibration curves construction

Aliquots from RDV and MFX standard solutions (100 µg/mL) ranging from (10–150) µg for RDV and from (10–100) µg for MFX were transferred into two separate sets of 10-mL volumetric flasks and completed to the mark with methanol.

### Ratio manipulating spectrophotometric methods (RD, ^1^DD and MC)

#### Optimization of experimental conditions

Several MFX concentrations (1, 2, 4, 5, 6, 7, 8, and 10 µg/ml) were tested using the general process under “3.1” for each method in order to determine the optimal divisor concentration for RDV.

Using the same general techniques, various RDV concentrations (1, 3, 5, 7, 8, 9, 11, 13, and 15 µg/ml) were tested for MFX.

The MFX spectrum (6.0 µg/ml) was split by the zero order absorption spectra (from 200 to 400 nm) for RDV. The RDV spectrum (8.0 µg/ml) was split by the zero order absorption spectra (from 200 to 400 nm) for MFX.

##### Ratio derivative method

Using Δλ = 4 nm and scaling factor = 10, the first derivative for each ratio spectrum was recorded. The calibration curve was created by plotting the amplitude values against each drug’s concentration after they were measured at 250 nm for RDV and 290 nm for MFX. Regression equations were computed.

##### Ratio difference method

The calibration curve was created by plotting the difference in peak amplitudes (ΔP) at the ratio spectra at 247 and 262 nm (ΔP_247 − 262 nm_) for RDV and at 299 and 313 nm (ΔP_299 − 313 nm_) for MFX against each drug’s concentration. Regression equations were computed.

##### Mean centering method

The ratio spectra (from 200 to 400 nm) were mean centered and the mean centered values were measured at 247 nm and 299 nm for RDV and MFX, respectively. The values were plotted against concentration of each drug to obtain the calibration curve. Equations for regression were calculated.

### For area under the curve method

We applied the AUC method using two selected wavelength ranges. The AUC values were calculated from the overlain spectra of the binary mixture, and Cramer’s Rule in conjunction with the Matrix Method was used to perform the analysis. For the mixture of two component X and Y, the total AUC over the given range is equal to the sum of the individual AUCs of X and Y. This relationship can be defined by two equations which correspond to the intervals of selected wavelengths.

Then, the system of equations was reformulated in terms of values of absorptivity and concentration. Using Cramer’s Rule, the absolute concentrations of X and Y were determined with the equations:

1 $$\:{\varvec{C}}^{\varvec{X}}=\frac{\left({{\varvec{a}}^{\varvec{Y}}}_{\varvec{\lambda\:}1-\varvec{\lambda\:}2}\:\:{\varvec{A}\varvec{U}\varvec{C}\:}_{\varvec{\lambda\:}3-\varvec{\lambda\:}4}\right)\:-\:\left({{\varvec{a}}^{\varvec{Y}}}_{\varvec{\lambda\:}3-\varvec{\lambda\:}4}\:\:{\varvec{A}\varvec{U}\varvec{C}}_{\varvec{\lambda\:}1-\varvec{\lambda\:}2}\:\right)}{\left({{\varvec{a}}^{\varvec{Y}}}_{\varvec{\lambda\:}1-\varvec{\lambda\:}2\:}\:{{\varvec{a}}^{\varvec{X}}}_{\varvec{\lambda\:}3-\varvec{\lambda\:}4}\right)\:-\:\left({{\varvec{a}}^{\varvec{Y}}}_{\varvec{\lambda\:}3-\varvec{\lambda\:}4}\:\:\:{{\varvec{a}}^{\varvec{X}}}_{\varvec{\lambda\:}1-\varvec{\lambda\:}2}\:\right)}$$

2 $$\:{\varvec{C}}^{\varvec{Y}}=\frac{\left({{\varvec{a}}^{\varvec{X}}}_{\varvec{\lambda\:}3-\varvec{\lambda\:}4}\:\:{\varvec{A}\varvec{U}\varvec{C}}_{\varvec{\lambda\:}1-\varvec{\lambda\:}2}\:\right)\:-\:\left({{\varvec{a}}^{\varvec{X}}}_{\varvec{\lambda\:}1-\varvec{\lambda\:}2}\:\:{\varvec{A}\varvec{U}\varvec{C}\:}_{\varvec{\lambda\:}3-\varvec{\lambda\:}4}\right)}{\left({{\varvec{a}}^{\varvec{Y}}}_{\varvec{\lambda\:}1-\varvec{\lambda\:}2}\:\:{{\varvec{a}}^{\varvec{X}}}_{\varvec{\lambda\:}3-\varvec{\lambda\:}4}\right)\:-\:\left({{\varvec{a}}^{\varvec{Y}}}_{\varvec{\lambda\:}3-\varvec{\lambda\:}4}\:\:\:{{\varvec{a}}^{\varvec{X}}}_{\varvec{\lambda\:}1-\varvec{\lambda\:}2}\:\right)}$$

In this work, the AUC values over the ranges 243–248 nm and 290–300 nm for remdesivir (RDV) and moxifloxacin (MFX) were also obtained. The values of absorptivity and AUC were incorporated in Eqs. (1) and (2) to compute the concentration of RDV and MFX respectively [55]. 

### For Q-analysis method

As per the Q-analysis method, we assign the first drug as X and the second drug as Y. This method depends on measuring the absorbance at two wavelengths; the isoabsorptive point (λ_iso_) and the λ_max_ of one of the components. Using relationships given by ax1 = ay1 and a path length L = 1, two fundamental equations were formed. By dividing and rearranging, expressions for the fraction of each component were found. In order to determine the absolute concentrations of X and Y, further rearrangement lead to the equations below:

3 $${C_x}\, = \,\{ ({Q_m} - {Q_y})/({Q_x} - {Q_y})\} \, \times \,({A_1}/{a_{x1}})$$

4 $${C_y}\, = \,\{ ({Q_m} - {Q_x})/({Q_y} - {Q_x})\} \, \times \,({A_1}/{a_{y1}})$$

Finally Eqs. (1) & (2) gives the absolute concentration value of drug ***X*** & ***Y***.

Absorbance values of RDV and MFX were quantified at 229 nm (λ_iso_) and 245 nm (λ_max_), and calibration graphs were generated. Equations (3) and (4) were employed to calculate the concentration of RDV and MFX by utilizing the absorptivity values of each component at the selected wavelengths [46, 56].

### For bivariate method

Bivariate calibration involves measuring two components (X and Y) at specific wavelengths to generate equations. These equations allow for the evaluation of concentration (CX and CY) [45]. The Kaiser approach uses slope values from linear regression equations to build sensitivity matrices (K) and estimate optimal pair of wavelengths for binary mixture determination [57, 58]. The absorbance was measured at 245 and 295 nm, and the corresponding regression equations were computed for both drugs. The obtained slopes and intercepts were used to calculate RDV and MFX concentrations [57]. 

### Methods applications

#### Laboratory prepared mixtures analysis

After preparing various ratios of laboratory-prepared mixtures, the spectra of these mixtures were measured and handled similarly to how the suggested procedures specify.

#### Pharmaceutical formulation analysis

##### RDV

Remdesivir-Eva Pharma^®^ four vials (100 mg/ 20 mL vial) were mixed correctly. In a 100 mL volumetric flask, precisely 2 mL of remdesivir (10 mg) were added. A volume of about 70 mL of methanol was then added to the flask. The solution was shaken vigorously for 15 min, and then it was sonicated for 30 min. A concentration of 100 µg/mL was obtained by adding methanol to the fluid until it reached 100 mL.

##### MFX

Ten Moxiflox^®^ tablets containing 400 mg of moxifloxacin hydrochloride (436.37 mg/tablet) were weighed and ground into a coarse powder. The powder, which is equivalent to 10 mg of MFX, was precisely weighed, then moved to a 100 mL volumetric flask, where methanol was added to bring the volume up to about 70 mL. The solution was sonicated for 30 min after being forcefully shaken for 15 min. After adding 100 mL of methanol, the fluid was filtered to achieve a concentration of 100 µg/mL.

##### Co-formulated RDV and MFX

A fixed-dose combination originated as RDV and MFX fixed-dose pills were not accessible in Egypt. Four finely powdered Moxiflox^®^ tablets (400 mg/tablet) and four Remdesivir-Eva Phama^®^ vials (100 mg/20 mL vial) were mixed together. After carefully adding two milliliters of this mixture (10 milligrams of RDV and 40 milligrams of MFX) to a 100 milliliter volumetric flask, the flask was filled with methanol to a capacity of about 70 milliliters. After 15 min of vigorous shaking, the mixture was subjected to a 30-minute sonication. Following the addition of 100 mL of methanol, the volume was filtered to achieve a concentration of 400 µg for MFX and 100 µg for RDV.

### The reported method

A previously reported green UV spectrophotometric method-the first derivative of ratio spectra-was selected for the quantitative analysis of remdesivir [15]. 

UV spectrophotometric technique was described for quantifying MFX in various medicinal formulations. The MFX concentration was measured at 296 nm in 0.1 N hydrochloric acid (pH 1.2) [36].

### Analysis of RDV and MFX in spiked human plasma

Various aliquots from the RDV and MFX standard solutions (10 µg mL^− 1^) were added to 15 mL centrifuge tubes that contained 1 mL of drug-free plasma. The protein was then denaturized by adding 3 mL of methanol. After two minutes of vortexing, the solutions in the centrifuge tubes were centrifuged for thirty minutes at 4000 rpm. Using a rotary evaporator, the protein-free supernatants were vacuum-evaporated until they were completely dry. The dry residue was dissolved in methanol, then placed in 10-mL volumetric flasks and completely filled with methanol [59, 60]. This method was performed with each medication at different concentrations within the working range. Both RDV and MFX contents have been evaluated using regression analysis.

## Results and discussion

In the present study, six simple and sensitive spectrophotometric methods were suggested for the selective quantitative determination of RDV and MFX without previous separation.

### Spectral characteristics

**Fig. 2:**
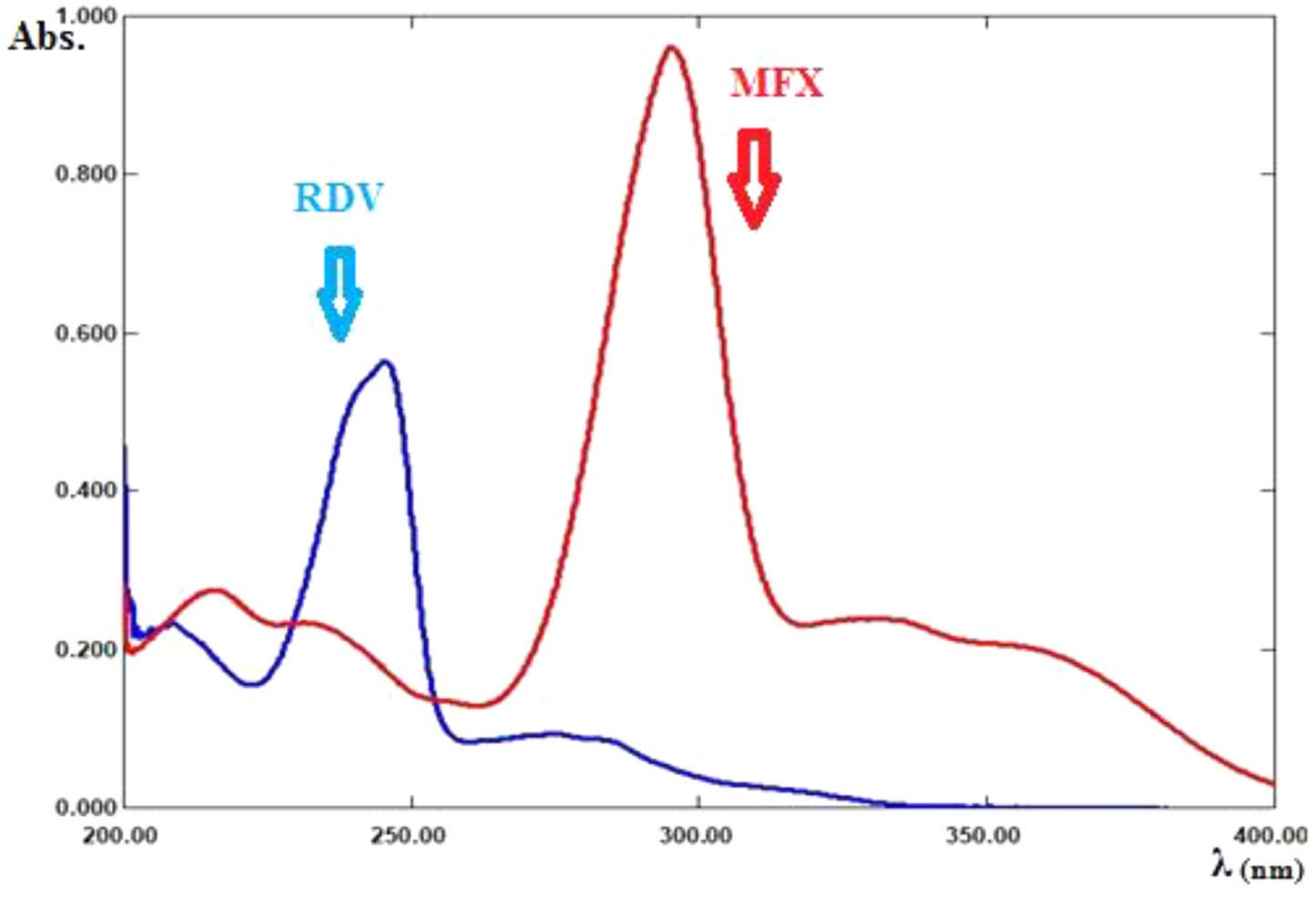
Zero order absorption spectra of RDV (8 µg/mL), and MFX (8 µg/mL)

The zero-order absorption spectra of RDV and MFX show severe overlap, as shown in Fig. 2 which does not permit direct determination of RDV and MFX presence of each other.

**Fig. 3:**
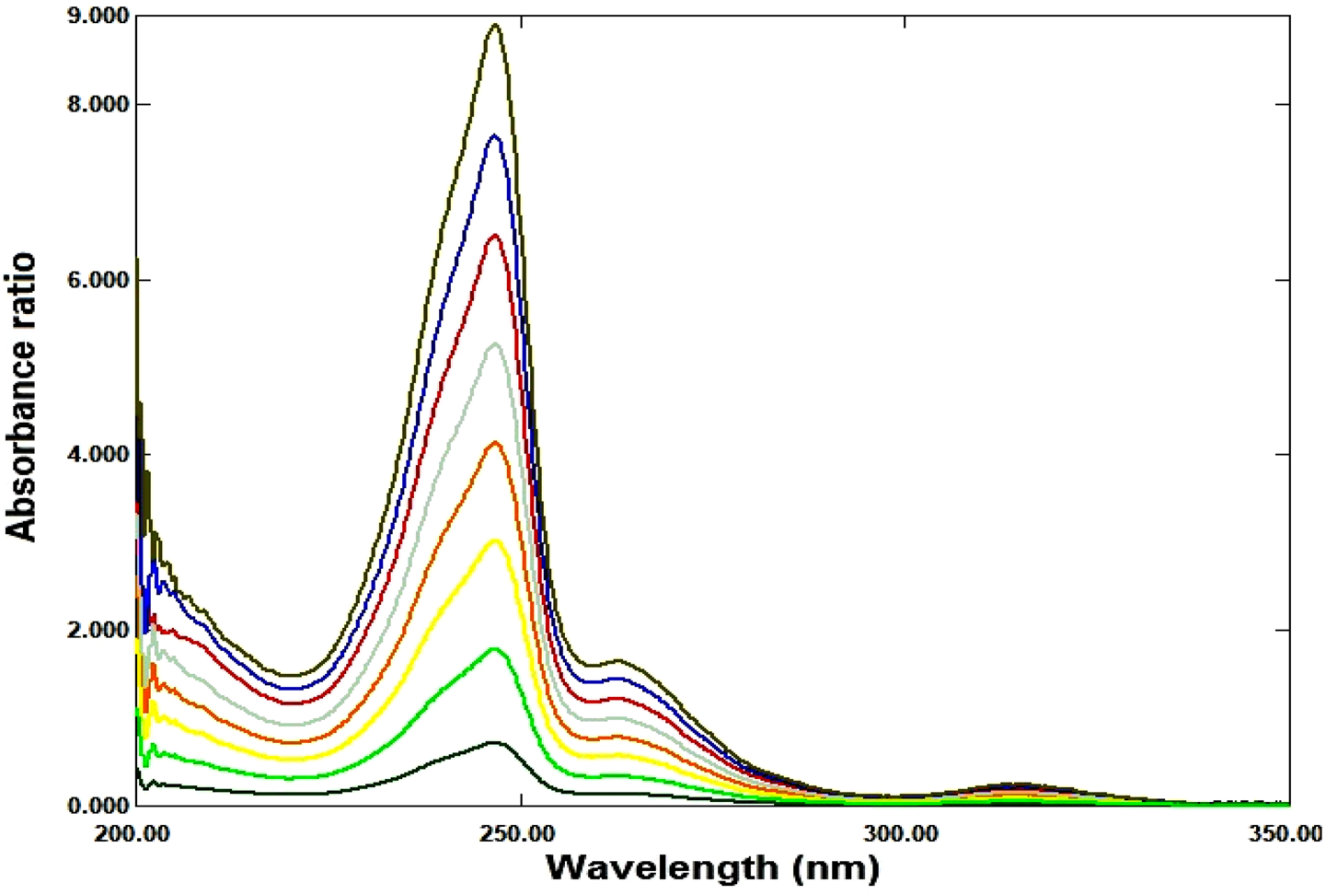
Ratio spectra of RDV at various concentrations (1–15 µg/ml) using 6 µg/ml of its MFX as a divisor

**Fig. 4:**
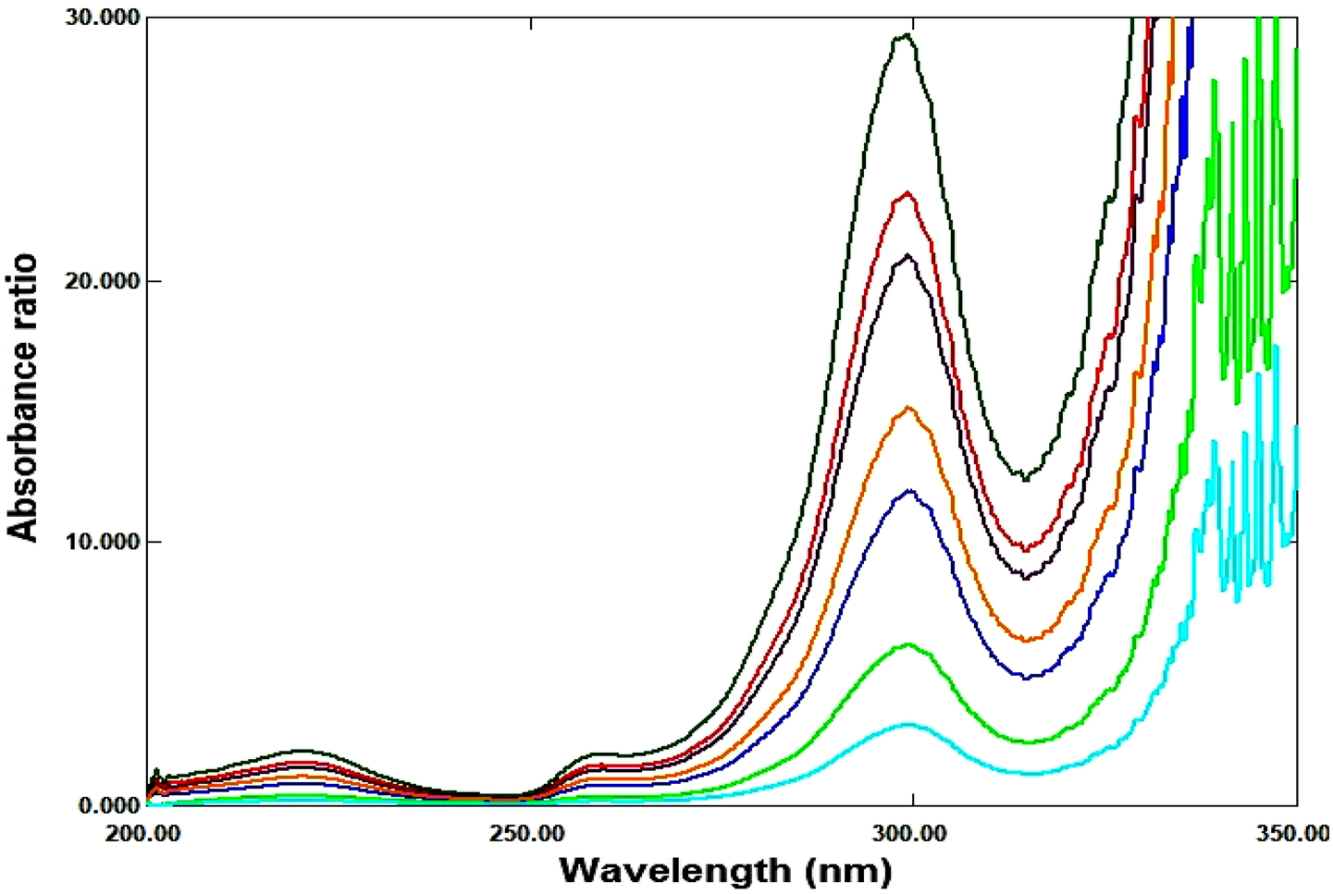
Ratio spectra of MFX at various concentrations (1–10 µg/ml) using 8 µg/ml of its RDV as a divisor

So, three proposed ratio methods are based on dividing the absorption spectra of each drug by the absorption spectrum of second drug, as a divisor, to get the ratio spectra, were used as shown in Figures (3, 4).

### Ratio derivative method

**Fig. 5:**
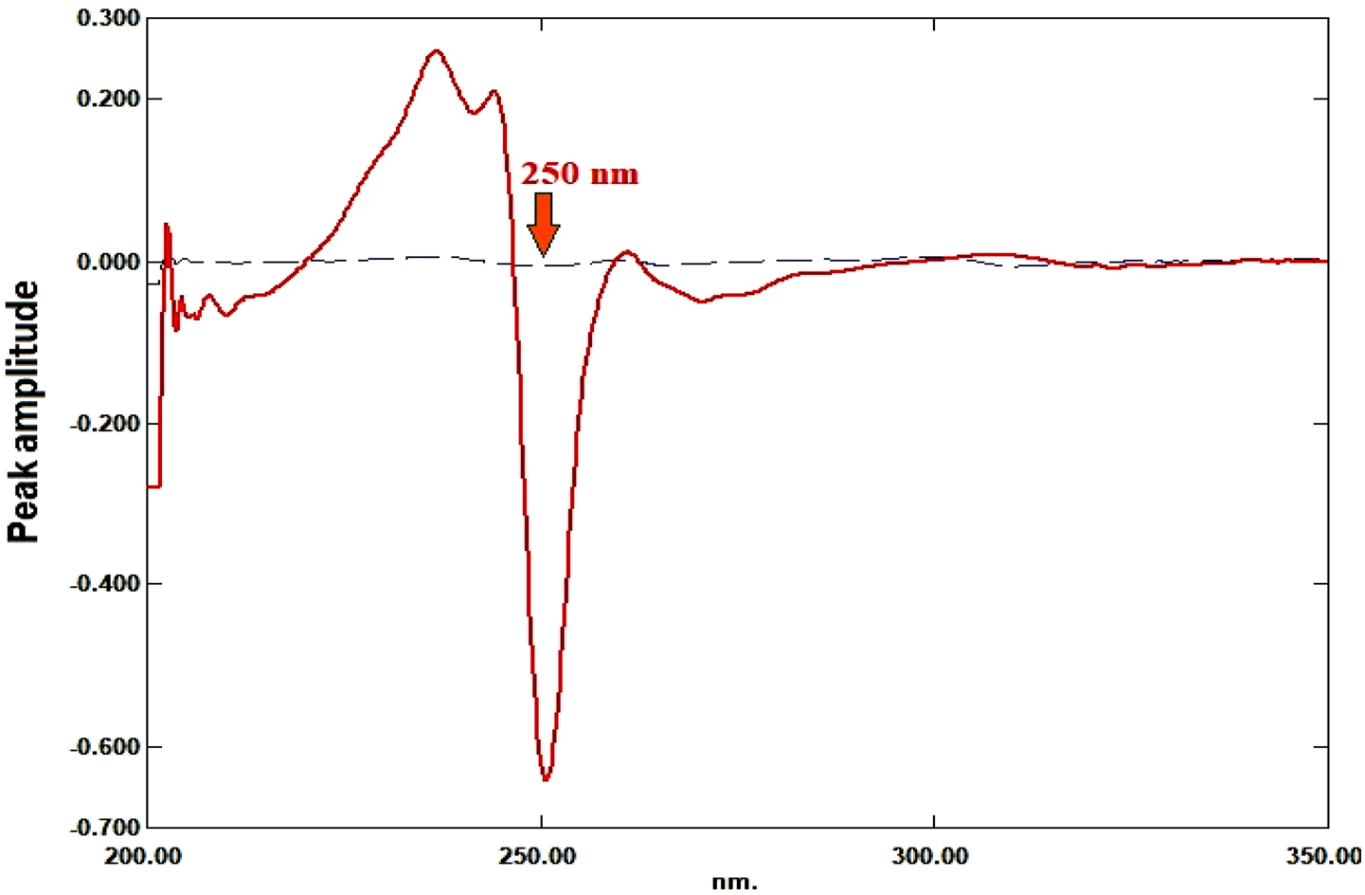
First derivative of the ratio spectra of RDV, 8 µg/mL, (ــــــ) and MFX, 6 µg/mL, (ـــ ــــ) using 6 µg/mL of MFX as a divisor

**Fig. 6:**
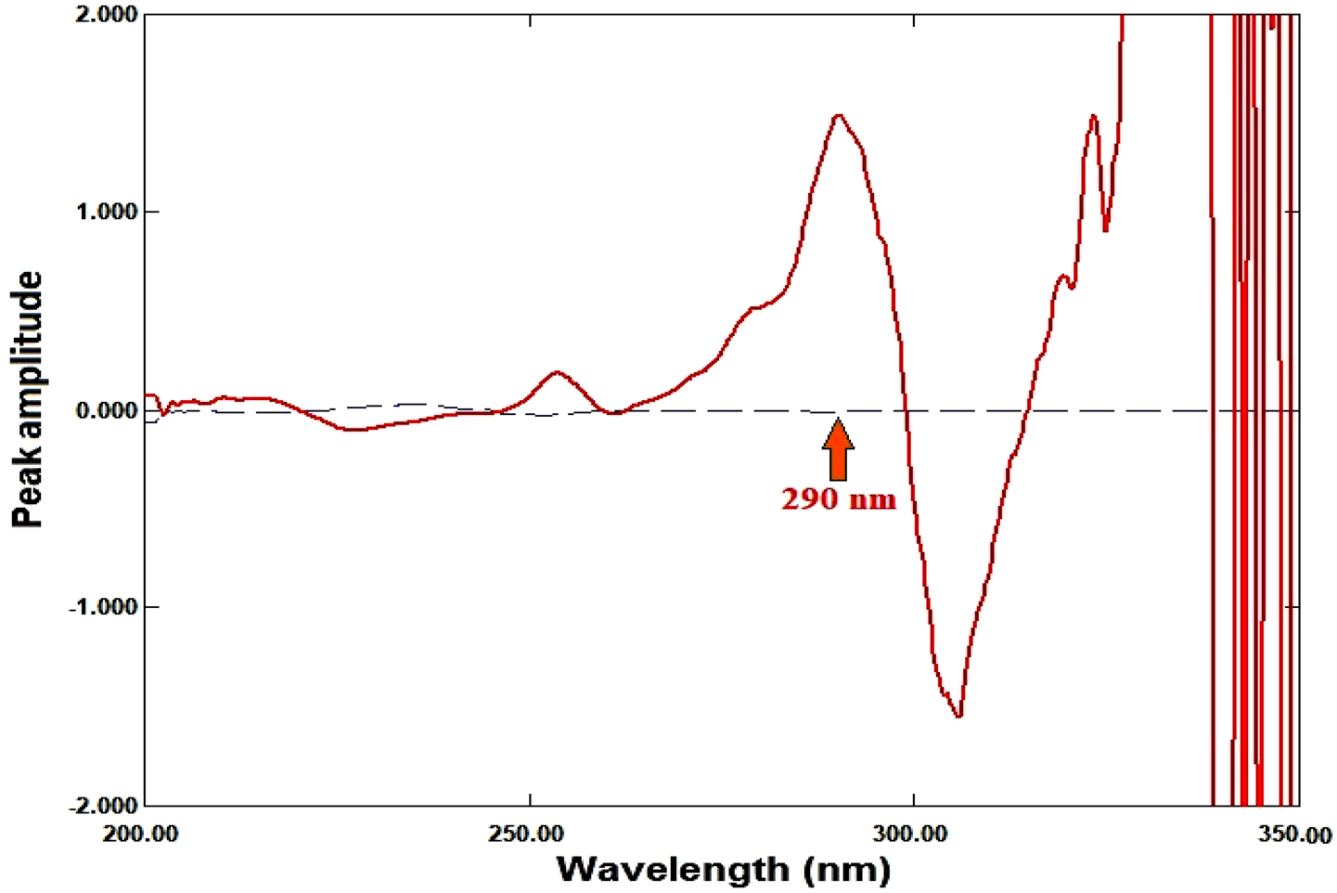
First derivative of the ratio spectra of MFX, 8 µg/mL, (ــــــ) and RDV, 8 µg/mL, (ـــ ــــ) using 8 µg/mL of RDV as a divisor

**Fig. 7:**
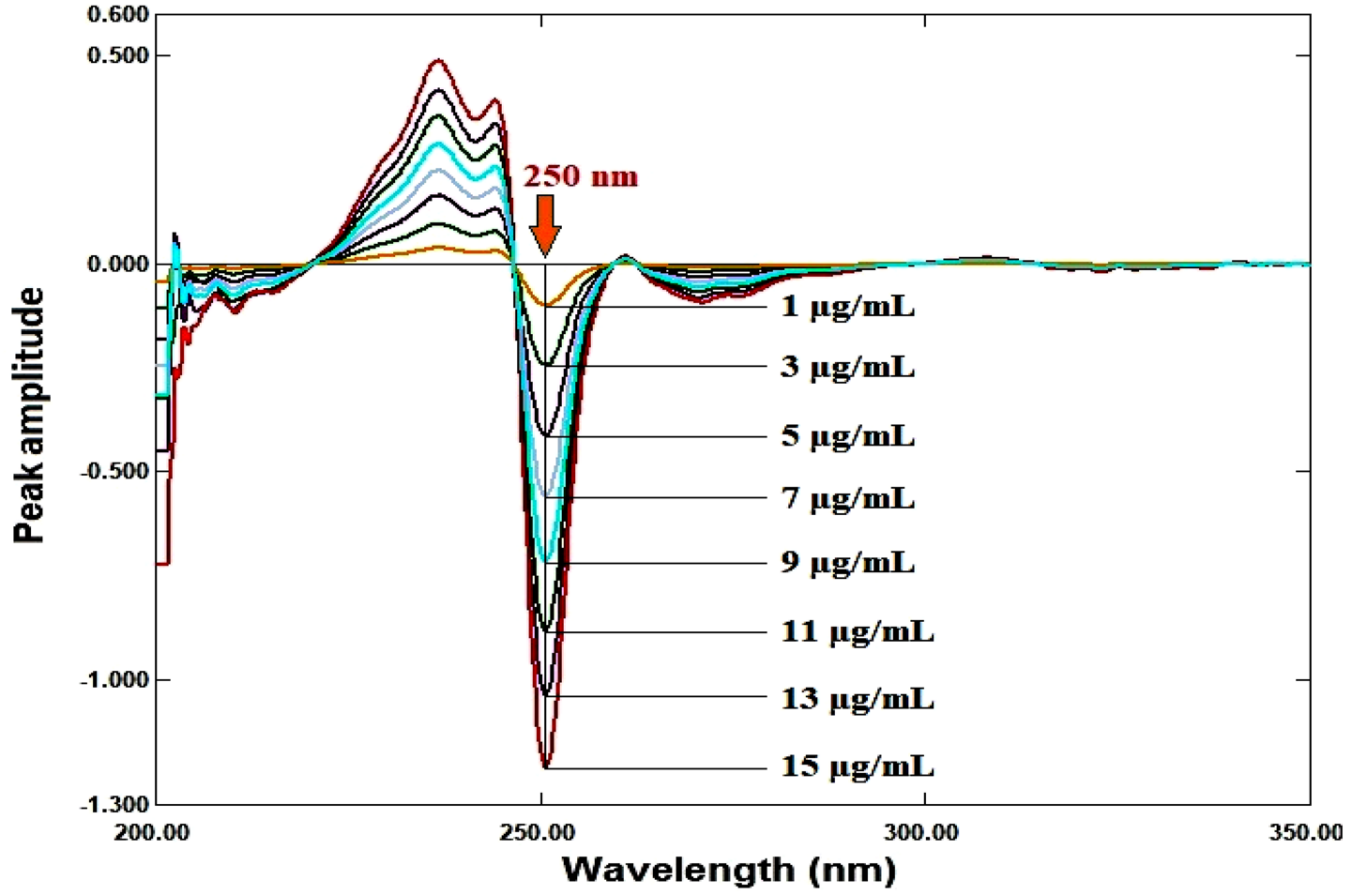
First derivative of the ratio spectra of RDV at various concentrations (1–15 µg/mL) using 6 µg/mL of MFX as a divisor

**Fig. 8:**
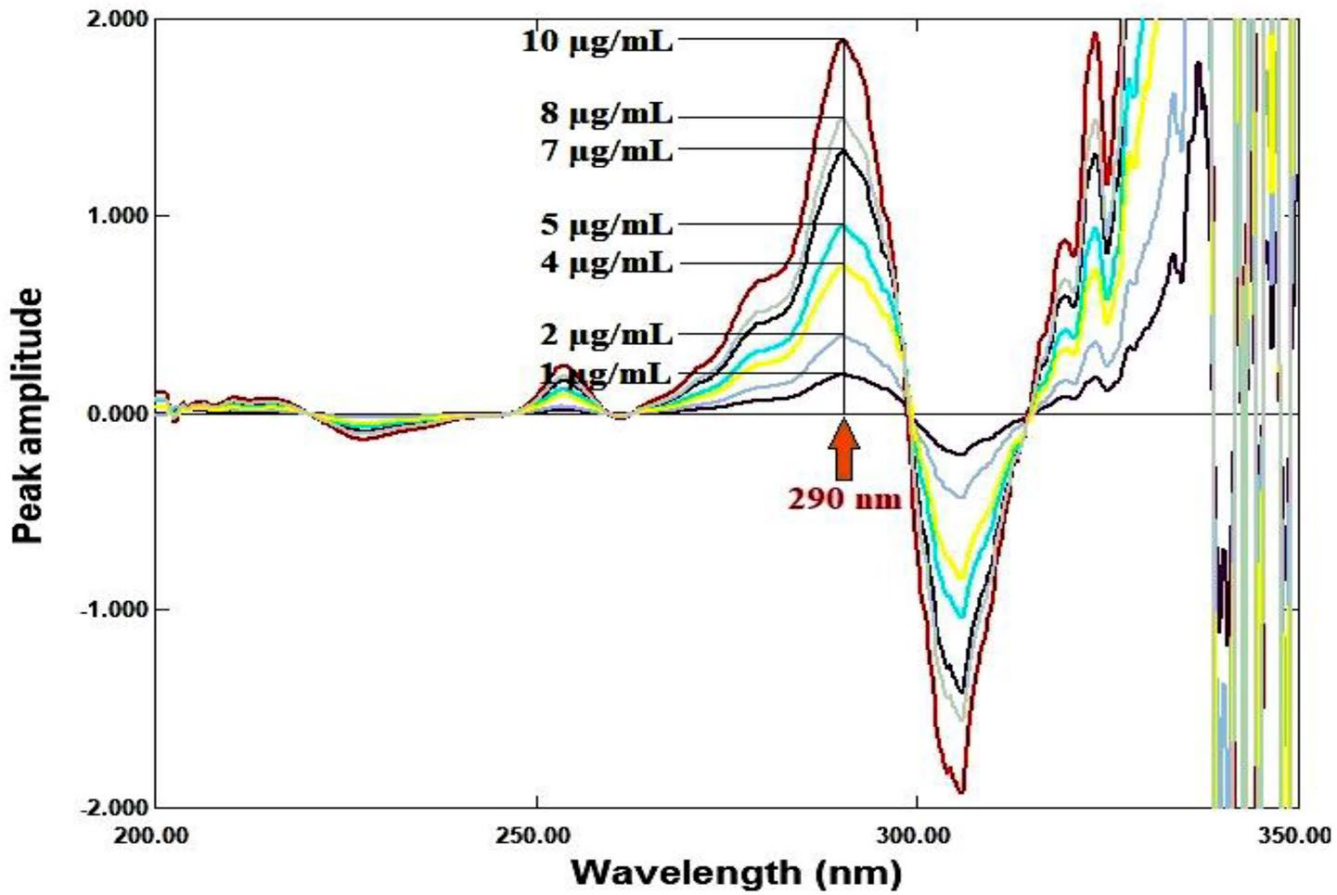
First derivative of the ratio spectra of MFX at various concentrations (1–10 µg/mL) using 8 µg/mL of RDV as a divisor

For RDV and MFX, respectively, the amplitudes of the first derivative of the ratio spectra at 250 nm and 290 nm are proportional to the concentrations of each medication independent of the other (divisor), as illustrated in Figures 5, 6, 7, 8.

### Ratio difference method

Without interference from the second medication (divisor), the ratio spectra’s peak amplitudes between (247–262) nm and (299–313) nm are proportionate to the RDV and MFX concentrations, respectively.

### Mean centering method

**Fig. 9:**
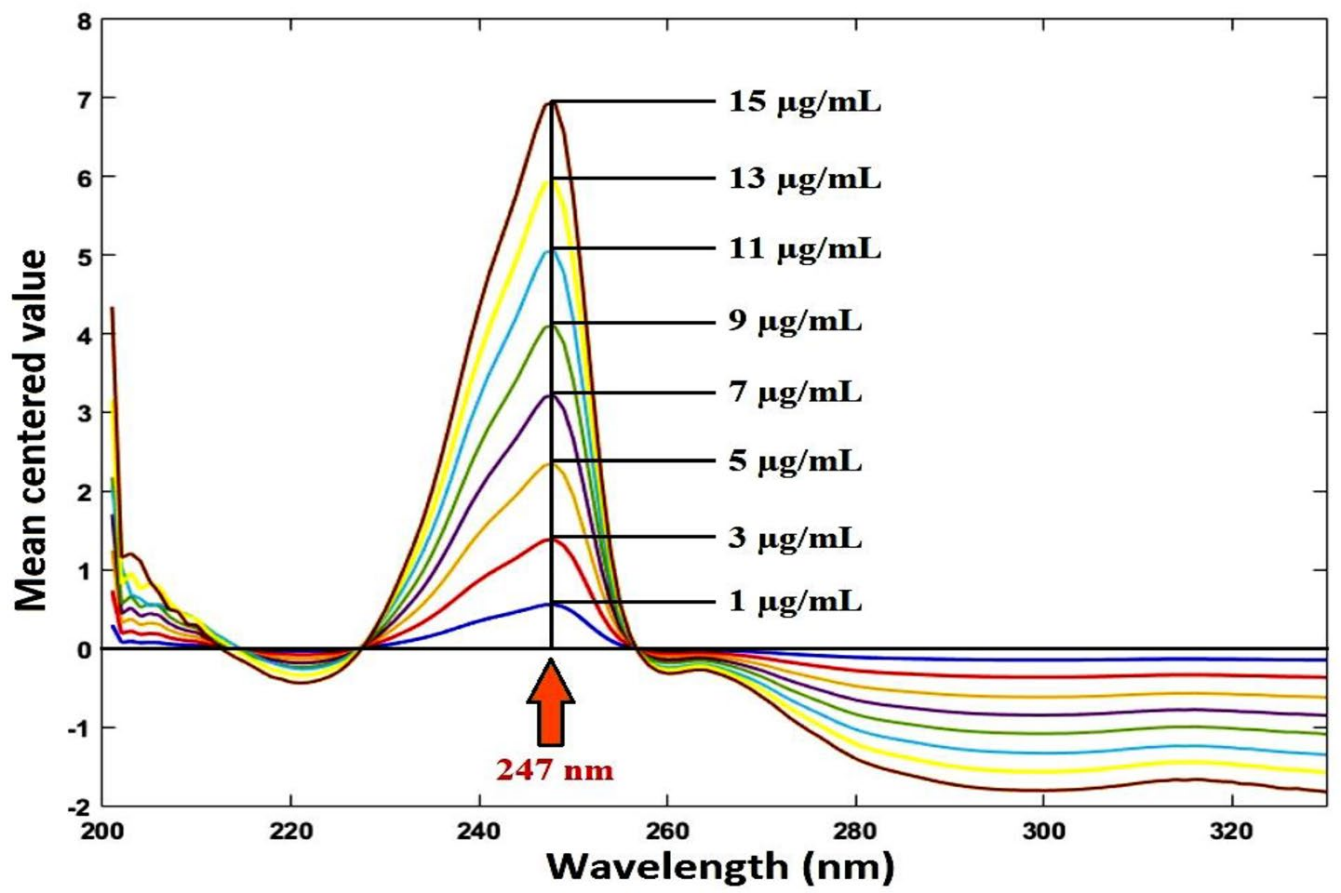
Mean centering of the ratio spectra of RDV at various concentrations (1–15 µg/ml) using 6 µg/mL of MFX as a divisor

**Fig. 10:**
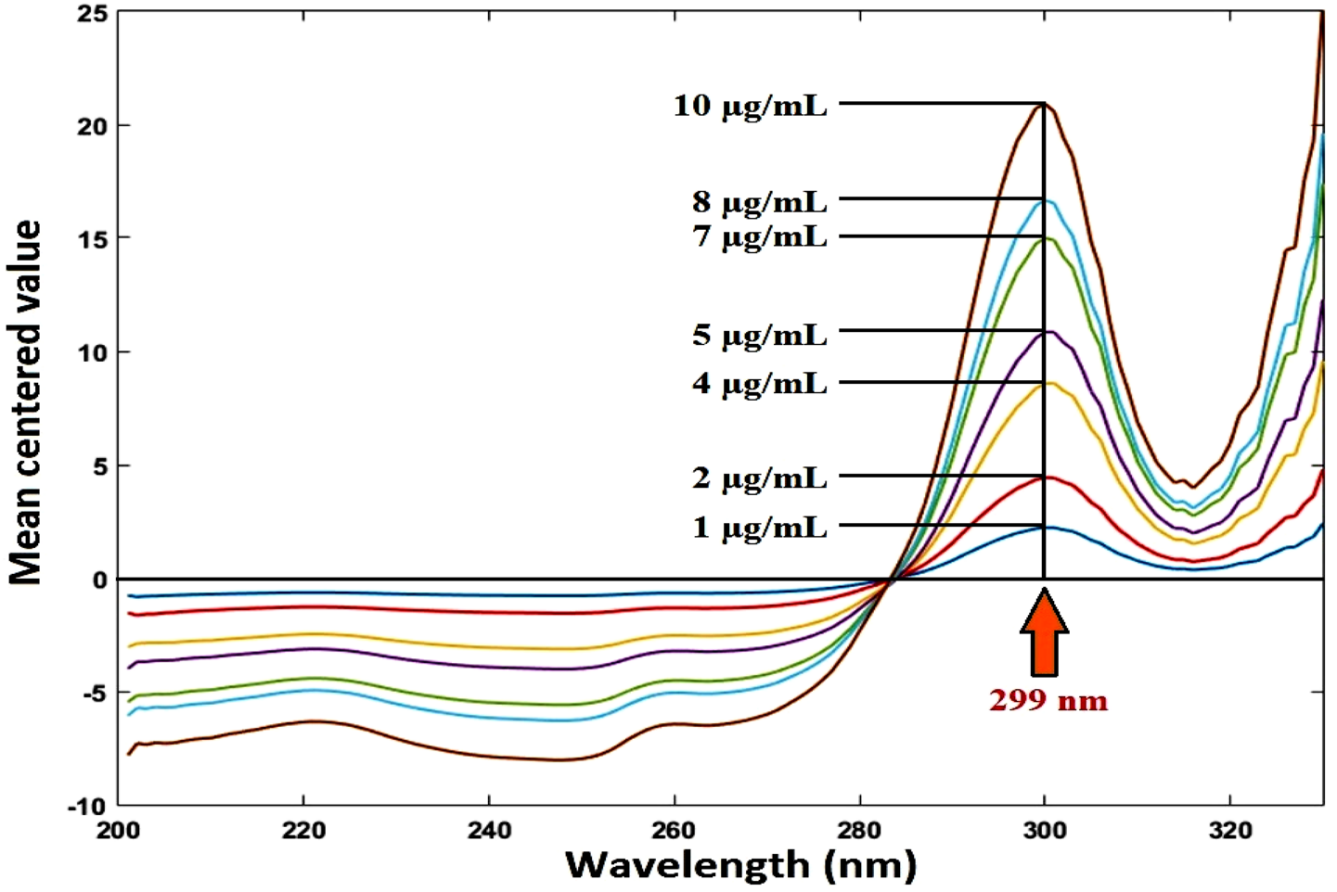
Mean centering of the ratio spectra of MFX at various concentrations (1–10 µg/ml) using 8 µg/mL of RDV as a divisor

Mean-centered ratio spectra were obtained. Without the other drug interfering, the mean centered values at 247 nm and 299 nm are proportional to the concentrations of RDV and MFX, respectively, as illustrated in Figures 9, 10.

Other three methods were also used including:

### Area under the curve method

**Fig. 11:**
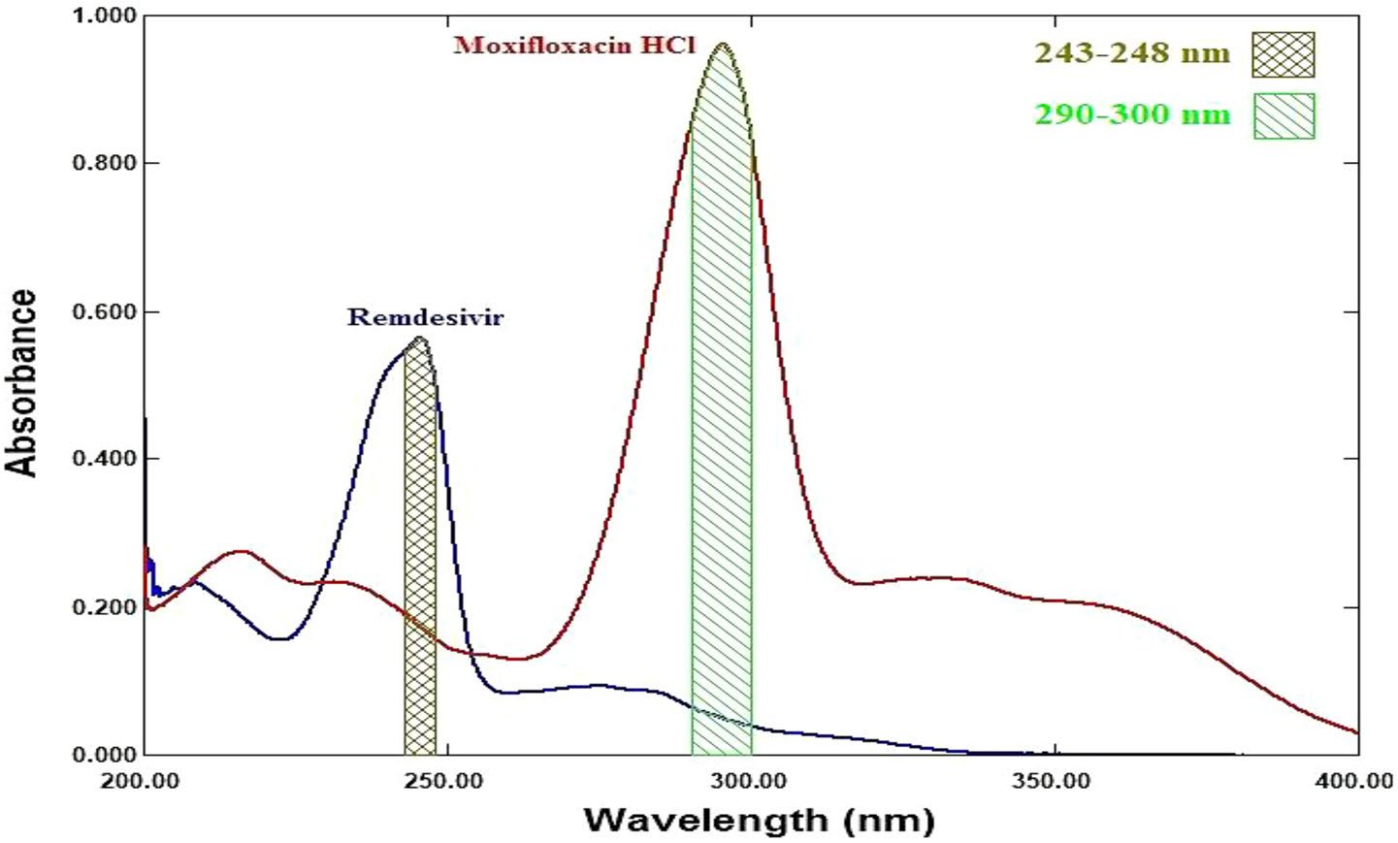
Zero order absorption spectrum of RDV (8 µg/mL) and MFX (8 µg/mL) showing area under the curve over the ranges (243–248) and (290–300) nm

Figure 11 lists the area under the curves for RDV and MFX, respectively, over the ranges of (243–248) and (290–300) nm. We created the calibration graphs and calculated the regression equations that connect the measured areas under the curve to the concentration of each component in µg/ml. The concentrations of RDV and MFX were determined using Eqs. (1) and (2), respectively, based on the absorptivity values and areas under the curve for each component at the chosen wavelength ranges.

### Q-analysis method

The absorption spectra of RDV and MFX showed isoabsorptive point at 229 nm, as shown in Fig. 2. The spectra show also isoabsorptive point at 254 nm which was not involved in the method due to the low sensitivity of the drugs at this wavelength. The absorbance values were measured at 245 nm (λ_max_ for RDV) and 229 nm (λ_iso_) in the range of 1–15 µg/ml and 1–10 µg/ml for RDV and MFX, respectively. Absorptivity values were determined for both RDV and MFX. The values and the absorbance ratio were used to calculate the concentration of RDV and MFX in their binary mixture using Eqs. (3) and (4), respectively.

### Bivariate method

To resolve RDV and MFX in their mixture using the bivariate approach, the absorbance of each component separately was measured at seven distinct wavelengths in the overlap region: 245, 255, 265, 275, 285, 295, and 305 nm. To make sure that there was a linear relationship between the absorbance and the appropriate concentration, the calibration curve equations and their accompanying linear regression coefficients were obtained. At the chosen wavelengths, every calibration curve displayed a reasonable linear determination coefficient (r2 > 0.9987). In accordance with the Kaiser approach, the sensitivity matrices (K) were computed using the slope values of the linear regression equations for both medications at the chosen wavelengths in order to determine the ideal pair of wavelengths at which the binary mixture was recorded. The slopes at 245 and 295 nm were selected for the analysis because they were found to provide the highest value of K (Table 1).

### Optimization of experimental conditions

When using ratio-based techniques, it was crucial to carefully select the divisor concentration. Various drug concentrations were tested as divisors; the best ones were 6 µg/ml and 8 µg/ml for RDV and MFX, as they produced the least amount of noise and produced better results in terms of selectivity.

### Method validation

The suggested techniques were successfully validated and produced satisfactory results in accordance with ICH criteria [61]. 

#### Linearity and range

The response of each drug for each method was plotted against the drug concentrations in µg/ml to create the calibration graphs for each drug for the three methods under the experimental circumstances indicated. The amplitudes of the first derivative of the ratio spectra at 250 nm and 290 nm for RDV and MFX, respectively, are the response for the ratio derivative method. The difference in peak amplitudes between the two chosen wavelengths (247–262) nm and (299–313) nm in the ratio spectra for RDV and MFX, respectively, is the response for the ratio difference method. The mean centered values of the ratio spectra at 247 nm and 299 nm for RDV and MFX, respectively, are the response for the mean centering approach. The area under the curve for the two chosen wavelength ranges, 243–248 nm (λ1-λ2) and 290–300 nm (λ3-λ4), is the response for the area under the curve method. Absorbance readings at 229 and 245 nm are the response for the Q-analysis approach. The absorbance readings at 245 and 295 nm provide the response for the bivariate technique. Table 2 displayed the regression data. The calibration graphs’ strong linearity was demonstrated by the coefficient of determination values.

#### Detection and quantitation limits

As shown in Table 2, the results of calculating LODs and LOQs values demonstrated the analytical sensitivity of the recommended techniques for the medications under investigation.

#### Accuracy and precision

The acquired good %R, as displayed in Table 2, indicated the accuracy of the suggested approaches. On the other hand, Table 2**’s** tiny percentage RSD values demonstrated the methodologies’ great precision.

#### Specificity

Different amounts of the drugs being studied were added to synthetic mixtures, which were then thoroughly mixed. Then, using the previously described basic approach of the process, the combinations were analyzed. Table 3 displays the excellent, satisfactory results that were obtained. As indicated in Table 4, the single dosage form of RDV and the co-formulated dosage form of MFX were identified in the laboratory using the recommended methodologies. Specificity was also evaluated by applying the traditional addition approach to pharmaceutical preparations that had previously been analyzed in order to determine the effect of the matrix on the identification of the two drugs. The data in Table 4 demonstrated that the recommended approach could assess the medication in a selected manner without influence from excipients.

### Pharmaceutical applications

The recommended techniques were used to measure the concentration of RDV and MFX in a single dosage form or in a dosage form that was co-formulated in the laboratory. The label claim that there were no indications of additive or excipient interference was well supported by the results of the conventional addition technique, which are displayed in Table 5. The results were compared to those obtained using the previously indicated approaches using statistics [15, 36]. The student’s t-test and F-test revealed no significant differences at the 95% confidence level [62], demonstrating the accuracy and precision of the suggested approach for evaluating the tested drug in its pharmaceutical dose form, as shown in Table 6.

### Spiked human plasma analysis

The new method was successful in monitoring RDV, and MFX at therapeutic levels in spiked human plasma samples due to the high sensitivity of the proposed approach. The plasma C_max_ for RDV, and MFX were 4420 ng/mL, and 3.56 mg/L, respectively, which exceed each drug’s LOD [63, 64]. Continuous monitoring of drug concentrations in plasma is crucial for optimizing therapeutic outcomes, ensuring drug efficacy, and avoiding toxicity—factors that ultimately contribute to lowering mortality rates. The newly developed techniques are appropriate for application in the study of the medications under research in human plasma due to their high sensitivity, as shown in Table 7.

### Method evaluation of greenness profiles

The analytical community’s foremost aim is to design procedures that address GC problems. GAC aims to promote a sustainable and eco-friendly approach to analytical chemistry, reducing the environmental effect while maintaining high accuracy and dependability. In recent years, various ways to evaluate greenness have emerged, including qualitative, semi quantitative, and quantitative methods. These approaches were created in line with the 12 GAC principles.

The provided approach’s environmental friendliness was assessed using the AGREE, ESA, and ComplexGAPI evaluation techniques. It’s interesting to note that every technique used to gauge greenness supported the idea that it is a very environmentally beneficial approach [65–67].

#### Assessment of greenness in compliance with ESA

A newly developed, comprehensive, semi quantitative instrument for evaluating the greenness of the approach [50]. It relies on deducting points—also known as penalty points—for analytical process characteristics that don’t match the 12 GAC ideas. An analysis that is more environmentally friendly has a higher score (closer to 100). Table 8 displays the ESA scores for the recommended approaches. With a high ESA score of 91 points, the recommended approach’s greenness was instantly apparent.

#### Assessment of greenness in compliance with complex GAPI

While ESA permits a basic assessment of greenness, Complex GAPI permits a more thorough semi-quantitative analysis [51]. Because it incorporates a hexagon-shaped region that depicts pre-analysis periods and stages, it is superior to the original GAPI measure. From sample collection and transportation to sample safety, storage, preparation, and analysis, this sophisticated tool manages every stage of the analytical process [51]. Simple pictogram software is provided by Complex GAPI. As demonstrated in Table 8, the suggested approach is more environmentally friendly because it demonstrated a lower E-factor (equal to 1), which suggests that the less waste produced, the greater and the more favorable the environmental impact.

Notwithstanding its advantages, ComplexGAPI’s focus is restricted to environmental standards, ignoring energy efficiency, waste reduction, and the integration of renewable resources. If ComplexGAPI is used excessively, it could lead to a limited and insufficient evaluation. This restriction can be addressed and a more thorough review can be obtained by combining ComplexGAPI analysis with quantitative evaluation techniques. In this integrated approach, ComplexGAPI and other supplementary tools will fill in research gaps and solve the limitations of utilizing a single evaluation method.

#### Assessment of greenness in compliance with AGREE

The most popular greenness assessment metric is AGREE [68]. All 12 of the GAC’s principles are included, making it comprehensive, adaptable (weighting is possible), easy to comprehend (a colored pictogram represents the end result), and simple to use (free software is accessible). The input parameters make reference to the 12 key principles and can be assigned different weights to offer some flexibility. The final score, which ranges from 0 to 1, is calculated using the 12 input parameters. A clock-like graph with a score and a color in the middle that represents the ultimate score is the end outcome. The range of possible scores is dusky green (= 1) to dusky red (= 0).

### Assessment of the presented method’s whiteness

The RGB12 tool, developed by Paweł-Nowak and his colleagues in June 2021, is a simple and quantitative whiteness evaluation tool that computes the sustainability level in terms of whiteness assessment and provides an easy-to-understand assessment of the methods in accordance with the 12 WAC considerations [54]. The twelve distinct algorithms that make up the RGB 12 algorithm are arranged under the general headings of four red, four green, and four blue. The most important GAC criteria, such as toxicity, the quantity of reagents and waste, energy consumption, and the effects on people, animals, and/or genetic alterations (GMOs), are covered by the green group (G1–G4). The validation parameters, such as the scope of application, accuracy, LOD, precision, and LOQ, are covered by the second group, often known as the red group (R1–R4). The third group, known as the blue group (B1–B4), deals with practical/economic needs, time efficiency, and cost efficiency. By summing the marks the technique receives for each of the three areas/colors using the RGB algorithm, the final actual value of “whiteness,” which evaluates how well the approach adheres to the principles of WAC, is calculated. Table 8 demonstrates that the proposed method has a high whiteness score of 91.7, indicating its benefits in terms of greenness, whiteness, sustainability, analytical efficacy, and economic and practical aspects [65–67].

### Assessment of the presented method’s blueness

The BAGI measure evaluates an analytical method’s “blueness” based on practical criteria, distinguishing it from greenness-focused tools [54]. BAGI evaluates an analytical process’s “blueness” by taking into account ten key factors, including the type of analysis, analytes, instrumentation, sample productivity, sample preparation requirements, number of samples examined per hour, reagents and materials, pre-concentration steps, automation level, and sample quantity. Each of the ten variables is scored from 1 (lowest) to 10 (highest). The final BAGI score is calculated as the geometric mean of all ten criteria. A higher BAGI score indicates that an analytical approach is more relevant, functional, and fits the intended goal. This study found that the proposed methods had high BAGI ratings of 71 as in Table 8, demonstrating their real-world applicability, high throughput, automation potential, and low operational costs. However, BAGI does not offer comprehensive sustainability quantification because it primarily concentrates on practical factors. As a result, we also used the RGB12 method to assess composite analytical sustainability while taking performance, usefulness, and greenness into account. The method’s value as a workable green chemistry substitute is confirmed by the consistently high tool ratings [65–67].

### Importance of the multi-tool evaluation approach

The study illustrated the advantages of assessing the sustainability of analytical techniques across several dimensions utilizing a number of complimentary tools (ComplexGAPI, AGREE, ESA, BAGI, RGB12). Greenness (impact on the environment), blueness (practicality/cost-effectiveness), whiteness (total sustainability, including performance and safety), and other parameters were evaluated holistically by these instruments. According to criteria including decreased toxicity, waste reduction, material efficiency, high throughput, automation possibilities, cheap cost, and outstanding analytical performance, the devised analytical techniques demonstrated superior sustainability.

The approaches were validated as highly sustainable and implementable best practices by the combined qualitative and quantitative evaluation, which also showed that they are in line with the environmental, economic, and performance domains of green analytical chemistry.

## Conclusion

This study proposes a complete set of six spectrophotometric methods developed for the simultaneous determination of RDV and MFX in difficult matrices such as pharmaceutical formulations and spiked plasma samples without any prior separation. The methods are based on solving the problem of spectral overlap using appropriate mathematical calculations, which proves that simple UV equipment, when used correctly, is as good as, if not better than, more sophisticated analytical equipment. The methods were rigorously validated according to ICH guidelines, confirming their accuracy, precision, specificity, and robustness, in addition to their analytical performance. Their sensitivity was adequate for the detection of RDV and MFX at clinically significant plasma concentrations, indicating their potential utility in therapeutic drug monitoring. One of the most important things about this work is that it uses advanced sustainability metrics. Using Eco-Scale, ComplexGAPI, AGREE, RGB12, and BAGI together made sure that the environmental impact, operational safety, and practical feasibility were all taken into account. The high scores for greenness, whiteness, and blueness show that the methods are in line with the ideas of Green and White Analytical Chemistry. These features make the methods particularly suited for use in routine quality control laboratories, especially in settings where access to sophisticated equipment is limited.

**Table 1:** The absolute values of the sensitivity matrix determinates calculated according to Kaiser’s method (k x 10^5^) for the mixture of RDV and MFX [57, 58]

λ/λ	245	255	265	275	285	295	305
245	0	948.57	1013.91	2186.31	5505.51	**8509.11**	4967.1
255		0	58.75	284.83	976.67	1642.28	952.33
265			0	169.04	702.96	1228.39	710.29
275				0	597.92	1230.15	703.49
285					0	770.63	413.09
295						0	-56.81
305							0

**Table 2:** Regression and validation data for determining RDV and MFX using the proposed methods

Parameters	RDV									MFX								
	^1^DD	RD	MC	AUC		Q-analysis		Bivariate		^1^DD	RD	MC	AUC		Q-analysis		Bivariate	
Wavelength (nm)	250	247–262	247	243–284	290–300	229	245	245	295	290	299–313	299	243–284	290–300	229	245	245	295
Linearity range (µg/mL)		1–15								1–10								
Slope	0.0771	0.4736	0.4556	0.348	0.058	0.071	0.028	0.021	0.071	0.1868	1.6476	2.0704	1.150	0.120	0.029	0.021	0.021	0.122
Intercept	0.0093	0.0470	0.0487	0.037	0.006	0.009	0.005	0.005	0.009	0.0083	0.2893	0.2893	0.066	0.005	0.001	0.005	0.005	0.002
LOD (µg/mL)	0.264	0.311	0.302	0.312	0.316	0.301	0.911	0.278	0.314	0.242	0.294	0.262	0.307	0.305	0.278	0.844	0.287	0.259
LOQ (µg/mL)	0.8005	0.941	0.916	0.946	0.957	0.273	0.828	0.844	0.951	0.734	0.892	0.793	0.931	0.925	0.287	0.868	0.868	0.785
Coefficient of determination (r^2^)	0.9997	0.9996	0.9996	0.9997	0.9995	0.9997	0.9995	0.9997	0.9996	0.9997	0.9995	0.9995	0.9996	0.9995	0.9997	0.9996	0.9996	0.9996
Accuracy (% R) ^a^	98.70	100.09	98.59	100.25		99.84		99.12		101.25	99.91	101.05	98.76		101.07		99.41	
**Precision (% RSD)** ^b^ - Repeatability - Intermediate precision	0.522 1.038	1.617 0.869	0.972 0.579	1.233 1.417		1.161 1.47		0.833 1.096		0.635 1.238	1.304 1.465	0.969 1.094	0.742 0.959		0.947 1.328		1.225 1.161	

^**a**^ Average of 9 determinations (3 concentrations repeated 3 times). ^**b**^ %RSD of 9 determinations (3 concentrations repeated 3 times)

**Table 3:** Analysis of laboratory prepared mixture of RDV and MFX by the proposed methods

Method	Laboratory prepared mixture (µg/mL)				% Recovery*	
	RDV		MFX		RDV	MFX
Ratio derivative	1		2		98.18	98.96
	1		4		100.78	99.13
	2		4		100.32	100.87
	2		8		99.03	101.02
	4		4		101.72	99.13
	Mean ± %RSD				100.01 ± 1.406	99.82 ± 1.033
Ratio difference	1			2	101.77	99.65
	1			4	100.08	101.55
	2			4	100.71	98.37
	2			8	101.67	101.08
	4			4	99.98	98.14
	Mean ± %RSD				100.84 ± 0.842	99.76 ± 1.547
Mean centering	1			2	101.38	100.79
	1			4	99.36	101.86
	2			4	100.42	98.72
	2			8	101.50	100.98
	4			4	99.48	98.12
	Mean ± %RSD				100.43 ± 1.007	100.10 ± 1.593
Area under curve	1	2			98.55	101.51
	1	4			100.36	98.18
	2	4			99.88	98.50
	2	8			100.06	99.02
	4	4			98.27	100.76
	Mean ± %RSD				99.42 ± 0.952	99.59 ± 1.468
Q- analysis	1	2			102.08	99.75
	1	4			99.71	100.80
	2	4			100.70	98.71
	2	8			101.10	101.32
	4	4			100.90	100.91
	Mean ± %RSD				100.90 ± 0.844	100.30 ± 1.053
Bivariate	1	2			98.48	98.76
	1	4			98.87	101.88
	2	4			101.63	101.30
	2	8			98.76	100.92
	4	4			99.72	100.22
	Mean ± %RSD				99.29 ± 0.887	100.62 ± 1.191

* Average of three determinations

**Table 4:** Determination of RDV and MFX in pharmaceutical preparations and co-formulated dosage form by the proposed methods

Method	Remdesivir-Eva 100 mg/vial		Moxiflox 400 mg/tablet		Co-formulated dosage form			
					RDV		MFX	
	Conc. (µg/mL)	% *R* *	Conc. (µg/mL)	% *R* *	Conc. (µg/mL)	% *R* *	Conc. (µg/mL)	% *R* *
Ratio derivative	1	100.77	4	98.06	1	99.48	4	99.13
	1.5	99.18	6	99.01	1.5	101.77	6	99.54
	2	98.38	8	98.41	2	100.97	8	100.62
	2.5	101.53	10	98.22	2.5	99.46	10	98.11
	Mean	99.97	Mean	98.43	Mean	100.42	Mean	99.35
	%RSD	1.144	%RSD	0.422	%RSD	1.142	%RSD	1.046
Ratio difference	1	100.72	4	101.87	1	100.08	4	101.70
	1.5	98.40	6	100.47	1.5	98.11	6	100.43
	2	100.30	8	101.16	2	99.66	8	99.73
	2.5	101.52	10	101.93	2.5	101.01	10	101.38
	Mean	100.23	Mean	101.36	Mean	99.72	Mean	100.81
	%RSD	1.323	%RSD	0.681	%RSD	1.213	%RSD	0.893
Mean centering	1	99.93	4	99.29	1	98.18	4	100.28
	1.5	98.67	6	100.25	1.5	99.25	6	98.18
	2	101.33	8	99.70	2	99.90	8	98.96
	2.5	100.82	10	99.13	2.5	98.45	10	99.32
	Mean	100.19	Mean	99.59	Mean	98.94	Mean	99.19
	%RSD	1.162	%RSD	0.501	%RSD	0.793	%RSD	0.877
Area under the curve	1	101.80	4	98.14	1	101.57	4	98.10
	1.5	98.76	6	98.47	1.5	99.60	6	98.26
	2	101.87	8	99.33	2	100.61	8	99.33
	2.5	98.16	10	98.29	2.5	98.89	10	98.16
	Mean	100.15	Mean	98.56	Mean	100.17	Mean	98.46
	%RSD	1.96	%RSD	0.539	%RSD	1.171	%RSD	0.59
Q-analysis	1	98.91	4	99.26	1	98.91	4	99.26
	1.5	98.65	6	100.19	1.5	98.92	6	100.71
	2	98.52	8	100.66	2	98.92	8	101.43
	2.5	100.49	10	99.73	2.5	99.86	10	100.24
	Mean	99.14	Mean	99.96	Mean	99.15	Mean	100.41
	%RSD	0.922	%RSD	0.601	%RSD	0.478	%RSD	0.904
Bivariate	1	99.01	4	99.42	1	99.08	4	98.19
	1.5	98.52	6	101.86	1.5	100.60	6	101.70
	2	99.81	8	101.21	2	100.49	8	101.82
	2.5	98.27	10	101.35	2.5	99.31	10	101.41
	Mean	98.90	Mean	100.96	Mean	99.87	Mean	100.78
	%RSD	0.680	%RSD	1.054	%RSD	0.788	%RSD	1.724

**Table 5:** Application of standard addition technique using the proposed methods

Method	Pharmaceutical (µg/mL)	Remdesivir		MFX	
		Pure added (µg/mL)	% R *	Pure added (µg/mL)	% R *
Ratio derivative	RDV 1 (1.01)* MFX 4 (3.93)*	1	98.18	3	99.34
		2	99.03	4	101.27
		3	98.01	5	100.07
	Mean		98.41		100.23
	%RSD		0.552		0.973
Ratio difference	RDV 1 (1.01)* MFX 4 (4.07)*	1	100.92	3	99.05
		2	99.98	4	100.57
		3	100.65	5	98.48
	Mean		100.52		99.36
	%RSD		0.486		1.086
Mean centering	RDV 1 (0.99)* MFX 4 (3.97)*	1	98.40	3	101.94
		2	100.23	4	99.06
		3	98.21	5	100.35
	Mean		98.95		100.45
	%RSD		1.129		1.436
Area under curve method	RDV 1 (1.02)* MFX 4 (3.92)*	1	100.88	3	99.79
		2	98.36	4	99.93
		3	100.56	5	98.06
	Mean		99.94		99.26
	%RSD		1.373		1.052
Q-analysis method	RDV 1 (0.99)* MFX 4 (3.97)*	1	100.31	3	101.80
		2	100.11	4	100.74
		3	98.86	5	101.51
	Mean		99.76		101.35
	%RSD		0.788		0.540
Bivariate method	RDV 1 (0.99)* MFX 4 (3.93)*	1	101.65	3	98.33
		2	98.95	4	101.75
		3	98.81	5	98.52
	Mean		98.80		99.53
	%RSD		1.602		1.927

^*^ Average of three determinations

**Table 6:** Statistical study and determination of RDV and MFX in pharmaceutical formulations using the proposed spectrophotometric and previously described methods

Parameters	RDV							MFX						
	^1^DD	RD	MC	AUC	Q-analysis	Bivariate	Reported method [15]	^1^DD	RD	MC	AUC	Q-analysis	Bivariate	Reported method [36]
Number of measurements	4	4	4	4	4	4	4	4	4	4	4	4	4	4
Mean % Recovery	100.42	99.73	98.94	100.15	99.14	98.90	99.79	99.35	100.81	99.19	98.56	99.96	100.96	99.49
% RSD	1.142	1.213	0.792	1.958	0.922	0.688	1.197	1.046	0.893	0.877	0.539	0.601	1.054	1.496
Variance	1.316	1.463	0.616	3.845	1.427	0.463	1.427	0.172	0.811	0.756	0.282	0.361	1.132	2.216
Student’s *t*-test * (2.447)	0.761	0.084	1.183	0.311	0.861	1.292	——	1.375	1.521	0.347	1.178	0.591	1.607	——
*F*-value* (9.277)	1.085	1.024	2.320	2.693	1.708	3.087	——	2.050	2.732	2.929	7.855	6.131	1.957	——

* The values in parenthesis are tabulated values of “*t* ” and “*F* ” at (p = 0.05) [69]

**Table 7:** Determination of RDV and MFX in spiked human plasma by the proposed methods

Method	RDV			MFX		
	Added (µg/mL)	Found* (µg/mL)	%Recovery	Added (µg/mL)	Found* (µg/mL)	%Recovery
Ratio derivative	1	0.956	95.59	1	0.925	92.45
	1	0.943	94.29	4	3.740	93.50
	2	1.877	93.84	8	7.621	95.26
	3	2.772	92.39	4	3.714	92.83
	13	12.538	96.45	5	4.757	95.14
	**Mean ± %RSD**		94.51 ± 1.665	**Mean ± %RSD**		93.84 ± 1.389
Ratio difference	1	0.952	95.23	1	0.966	96.61
	1	0.920	92.06	4	3.758	93.97
	2	1.891	94.59	8	7.579	94.75
	3	2.740	91.36	4	3.836	95.92
	13	12.426	95.59	5	4.697	93.95
	**Mean ± %RSD**		93.77 ± 2.055	**Mean ± %RSD**		95.04 ± 1.251
Mean centering	1	0.931	93.13	1	0.953	95.28
	1	0.920	92.03	4	3.801	95.03
	2	1.827	91.34	8	7.598	94.98
	3	2.823	94.11	4	3.693	92.32
	13	12.141	93.39	5	4.665	93.29
	**Mean ± %RSD**		92.80 ± 1.192	**Mean ± %RSD**		94.18 ± 1.385
Area under the curve	1	0.933	93.31	1	0.961	94.66
	1	0.946	94.63	4	3.771	93.98
	2	1.928	96.40	8	7.773	95.86
	3	2.854	95.13	4	2.809	91.52
	13	12.093	93.02	5	3.694	92.62
	**Mean ± %RSD**		94.50 ± 1.461	**Mean ± %RSD**		93.73 ± 1.816
Q-analysis method	1	0.948	94.75	1	0.923	92.33
	1	0.961	96.09	4	3.756	93.89
	2	1.898	94.91	8	7.501	93.76
	3	2.856	95.21	4	3.728	93.20
	13	12.268	94.37	5	4.626	92.52
	**Mean ± %RSD**		95.07 ± 0.683	**Mean ± %RSD**		93.14 ± 0.759
Bivariate method	1	0.94	93.71	1	0.96	96.03
	1	0.93	92.80	4	3.81	97.04
	2	1.93	94.07	8	7.53	96.40
	3	2.76	92.06	4	3.72	93.04
	13	12.39	95.31	5	4.86	97.26
	**Mean ± %RSD**		94.06 ± 1.897	**Mean ± %RSD**		95.12 ± 1.729

**Table 8:**
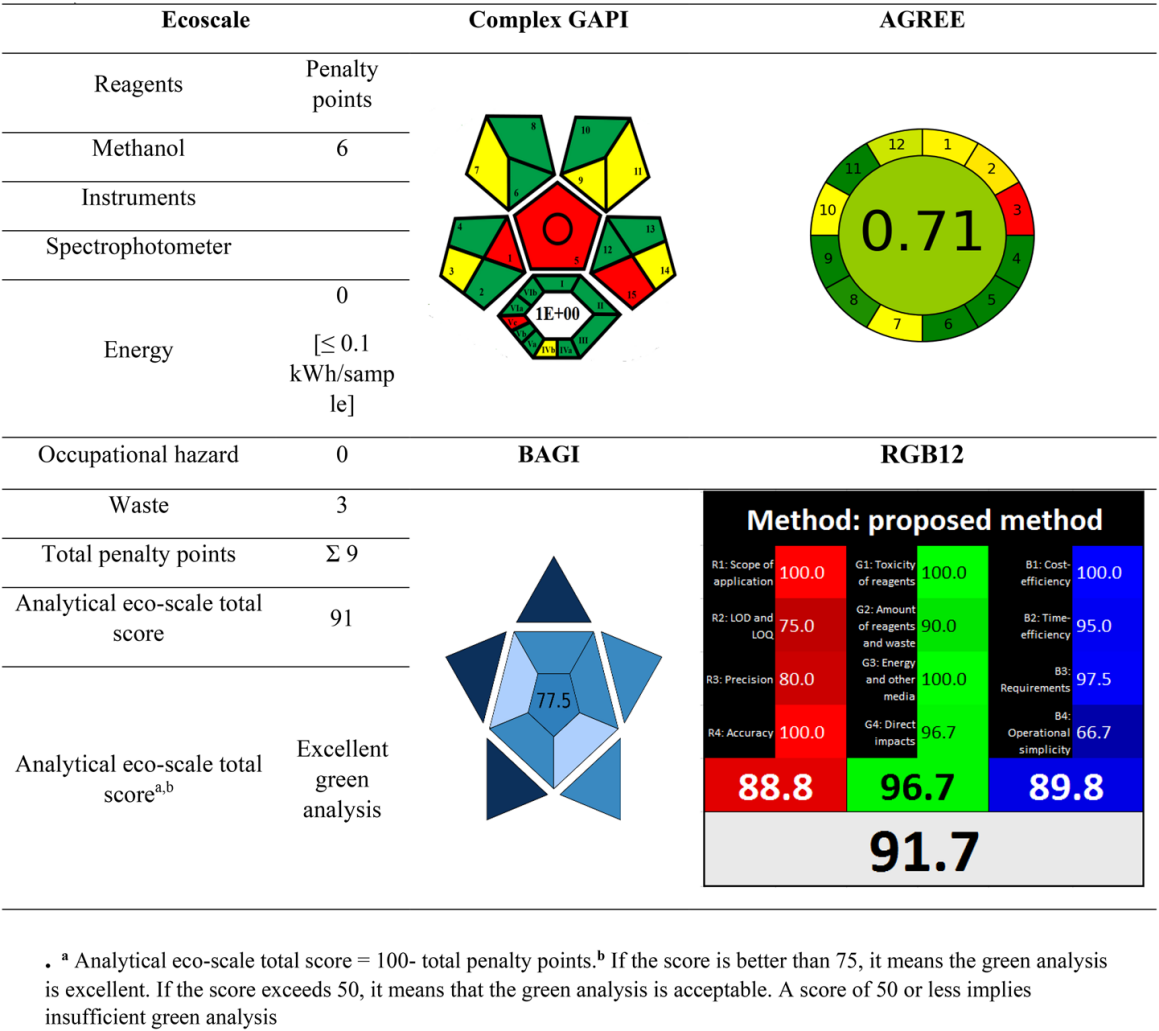
Greenness, whiteness, and blueness assessment of the proposed spectrophotometric approach according to ESA, complexgapi, AGREE, BAGI and RGB12 tools

## Data Availability

The datasets used and/or analyzed during the current study are available from the corresponding author on reasonable request.
